# The Role of Vitamin Deficiency in Liver Disease: To Supplement or Not Supplement?

**DOI:** 10.3390/nu13114014

**Published:** 2021-11-10

**Authors:** Anna Licata, Maddalena Zerbo, Silvia Como, Marcella Cammilleri, Maurizio Soresi, Giuseppe Montalto, Lydia Giannitrapani

**Affiliations:** 1 Internal Medicine & Hepatology Section, Department of Health Promotion Sciences, Maternal and Infant Care, Internal Medicine and Medical Specialties—PROMISE, University of Palermo Medical School, 90127 Palermo, Italy; maryzerbo@libero.it (M.Z.); silvia.como29@gmail.com (S.C.); marcella.cammilleri@gmail.com (M.C.); maurizio.soresi@unipa.it (M.S.); giuseppe.montalto@unipa.it (G.M.); lydia.giannitrapani@unipa.it (L.G.); 2 Institute for Biochemical Research and Innovation, National Research Council (CNR), 90146 Palermo, Italy

**Keywords:** vitamins, chronic liver disease, micronutrients, vitamin supplementation

## Abstract

Over the past few years, growing interest has been shown for the impact of dietary requirements and nutritional factors on chronic diseases. As a result, nutritional programs have been reinforced by public health policies. The precise role of micronutrients in chronic liver disease is currently receiving particular attention since abnormalities in vitamin levels are often detected. At present, treatment programs are focused on correcting vitamin deficiencies, which are frequently correlated to higher rates of comorbidities with poor outcomes. The literature reviewed here indicates that liver diseases are often related to vitamin disorders, due to both liver impairment and abnormal intake. More specific knowledge about the role of vitamins in liver disease is currently emerging from various results and recent evidence. The most significant benefits in this area may be observed when improved vitamin intake is combined with a pharmacological treatment that may also affect the progression of the liver disease, especially in the case of liver tumors. However, further studies are needed.

## 1. Introduction 

Vitamin deficiency is a common finding in chronic liver diseases, such as alcoholic liver disease (ALD), non-alcoholic fatty liver disease (NAFLD), cirrhosis and hepatocellular carcinoma (HCC).

ALD poses a considerable global health problem, causing chronic liver damage and a severe imbalance of micronutrients due to the resulting malnutrition. Recently, many trials have been carried out to understand the pathophysiological mechanisms of alcohol toxicity and the severe deficiency of micronutrients, such as vitamins and trace elements like minerals, which could be considered a target for supplementation [1].

NAFLD is the most common cause of liver disease in the western world [2]. In some patients, it can progress to NASH (non-alcoholic steatohepatitis), thus increasing the risk of evolution to cirrhosis and HCC [3]. Recently, there has been trend of rapid growth in NAFLD prevalence in developing countries. Worldwide prevalence is between 3% and 45% in the general population [4] and it is higher in obese and diabetic individuals [2]. Despite it being widespread, the pathogenesis of NAFLD is still not fully understood, and there is currently no targeted therapy [5]. The literature shows that also patients with NAFLD/NASH present micronutrient deficiency, which may contribute to the actual disease pathogenesis, especially in the case of oxidative stress.

Similarly, patients with cirrhosis and HCC present several important vitamin deficiencies.

Supplements of vitamins and oligo-elements could represent a supportive therapy to overcome deficiencies in this category of patients. However, despite the numerous studies to date, the clinical data on liver disease patients remain inconclusive.

This review aims to update the reader on the role of vitamins in chronic liver disease, focusing on the etiology and staging of liver disease in both alcoholic and non-alcoholic fatty liver disease, cirrhosis and HCC. Lastly, liver transplant conditions will also be considered, taking into account the degree of immunodeficiency that this condition entails. Our practical goal, however, will be to indicate a clear and appropriate micronutrient supplementation strategy whenever clinical conditions require it.

## 2. Physiological Role of Vitamins 

Vitamins are essential micronutrients involved in several biological functions. Many important stages of their metabolism, storage and activation occur in the liver, so it would be expected that chronic liver diseases may impair these processes [6]. Indeed, most vitamin levels appear to be reduced in many liver diseases. Because of their role, it is important to assess what happens in conditions of vitamin deficiency and whether there could be a therapeutic role for their supplementation in treating some hepatic disorders. 

Vitamin A plays a crucial role in various physiological processes, from embryogenesis to cell proliferation and differentiation, vision, immune regulation and glucose and lipid metabolism in the liver and adipose tissue. The liver contributes to vitamin A absorption thanks to the production of bile and its distribution to peripheral tissues via the retinol binding protein 4 (RBP4); it hosts the largest body supply of vitamin A, in the form of retinyl esters, in the hepatic stellate cells (HSCs). Vitamin A performs most of its functions through retinoic acids, which activate transcriptional networks by means of retinoic acid receptors and retinoid X receptors [7]. 

The B-vitamin group consists of eight water-soluble constituents that perform essential, closely interrelated roles, acting as co-enzymes in a vast array of catabolic and anabolic enzymatic reactions [8], and the group is also an intermediary for the biosynthesis of numerous key compounds, including amino acids, fatty acids and pyrimidines. The eight constituents are thiamine (B_1_), riboflavin (B_2_), niacin (B_3_), pantothenic acid (B_5_), pyridoxine (B_6_), biotin (B_7_), folate (B_9_) and cobalamin (B_12_) [9]. These essential nutrients are primarily supplied by food in the diet [10]. Several vitamin B coenzymes also contribute to cellular processes, such as the “folate cycle” and the “methionine cycle”, which are both essential to cell function [11].

Vitamin C, also known as ascorbic acid, is a simple low-molecular-weight carbohydrate, ubiquitous in nature and water-soluble, with an enediol structure that makes it an electron donor. Humans, among some other species, cannot synthesize vitamin C and therefore rely on food in the diet to acquire the necessary supply: fruit and vegetables are good sources of vitamin C, and about 90% of its daily intake in the general population comes from citrus fruit, kiwis, mangos, tomatoes and peppers. The optimum intake is about 200 mg/day for most of the adult population [12]. Vitamin C acts as a reductant as it donates an electron to a substrate. Therefore, its biological role is related to the reduced form, ascorbate, and it performs both enzymatic and non-enzymatic functions. It acts as a cofactor for the hydroxylation of lysine and proline residues in the synthesis of collagen [13]. In addition to its roles in these enzymatic processes, vitamin C is a powerful antioxidant with the ability to reduce or scavenge many physiologically relevant free radicals and reactive oxygen species (ROS), protecting biological macromolecules from oxidative damage, which might otherwise contribute to the initiation and progression of several acute and chronic diseases [14]. 

Vitamin D is a fat-soluble hormone with an important role in the regulation of bone metabolism and calcium homeostasis, but it also appears to have a role in hepatic fibrogenesis [15]. It is obtained from exposure to sunlight, foods in the diet, and some health supplements [16]. It undergoes its first biotransformation in the liver via hydroxylation in position 25 (25-hydroxyvitamin D, 25 (OH) D), while the second one, 1α-hydroxylation, occurs in the kidneys. Since the first important stage occurs in the liver, chronic liver diseases may be associated with its impaired metabolism [6]. Vitamin D receptor (VDR) also plays a role in metabolizing vitamin D; it is present in high quantities in hepatocytes, HSCs, and sinusoidal, endothelial and Kupffer cells, which can become potential targets when it increases due to inflammation [17].

Vitamin E includes a group of eight liposoluble compounds, including four tocopherols and four tocotrienols, of which alfa-tocopherol is the most biologically active. Tocopherol plasma levels are closely related to lipid plasma levels. Primarily, it is an antioxidant liposoluble compound, acting as a free radical, inactivating cellular membranes by which it is incorporated. Moreover, vitamin E enhances cell-mediated immunity, influences gene expression and regulates enzymatic activity by inactivating protein kinase C (PKC) and smooth muscle growth. Several studies have reported lower levels of liposoluble vitamins in chronic liver disease, especially in alcoholic and cholestatic liver diseases (such as primary biliary cholangitis) and HCC. Vitamin E supplementation reverses the neurological abnormalities of children with chronic cholestasis, whereas its severe deficiency becomes difficult to correct, especially in adults with advanced liver disease requiring liver transplantation [18].

Vitamin K includes a group of liposoluble vitamers, which in their biologically active forms take part in many calcium-dependent protein activities involved in bone tissue homeostasis and coagulation pathways. Vitamin K_1_, also known as phylloquinone, can be found mainly in green plant sources. In the human body, it is turned into its biologically active form (vitamin K_2_ or menaquinone) by intestinal bacteria living in the distal small intestine and colon. Vitamin K acts as a co-factor in the carboxylation of glutamate residues to gamma-carboxyglutamate in many proteins [19]. These modified remnants are situated in specific protein domains (Gla domains), which allow vitamin K-dependent proteins to bind calcium, resulting in protein activation to maintain their activities on blood coagulation and bone homeostasis: pro-coagulation factors II (prothrombin), VII, IX and X; coagulation inhibitor proteins C and S; osteocalcin; and matrix Gla protein. Vitamin K is absorbed in the small intestine and is stored in the liver and adipose tissue. It is rapidly metabolized, so cholestatic diseases leading to malabsorption are related to deficiencies of fat-soluble vitamins [20].

Antioxidants play a key role in chronic liver disease, as they are substances that inhibit the oxidation of any biomolecules [21], which neutralize the harmful effects caused by free radicals maintaining the homeostatic redox state. Antioxidants can be endogenous (for example, glutathione–GSH, superoxide dismutase, SOD_2_) or could be taken up from foods in the diet, such as olive oil. Anthocyanins, lycopene, coenzyme Q_10_, flavonoids, β-carotene, selenium and catechins are substances normally present in a diet with high antioxidant activity.

Vitamins and antioxidants are commonly involved in all metabolic processes of the organism. While analyzing the therapeutic need for their supplementation in chronic liver diseases of alcoholic and metabolic etiology, we will investigate the individual roles of vitamins B, D and E in alcoholic disease; those of vitamins C, E, A and D in NAFLD; and those of vitamins A, D and K in advanced cirrhosis and HCC. Lastly, the role of vitamin D will be also considered in transplanted immunocompromised patients.

## 3. Alcoholic Liver Disease: Malnutrition and Supplementation 

Alcohol use is one of the main causes of preventable disease and liver-disease-associated mortality worldwide. Alcohol is also a frequent co-factor in patients with other types of hepatic disorders, such as viral infection, where it accelerates hepatic fibrosis [22]. Alcohol consumption can cause a wide spectrum of liver damage which, through macrovesicular steatosis and steatohepatitis, can lead to cirrhosis and hepatocellular carcinoma [23]. The incidence of alcohol disease is increasing, especially among the younger generations, which raises concerns [24]. Globally, excessive alcohol consumption is responsible for approximately half of all liver diseases [25]. In detail, the estimated global mortality from alcoholic liver disease was 7.6% of all deaths [26]. From 2005 to 2015, the global incidence of alcoholic liver cirrhosis and HCC increased, rapidly rising from 9.8% to 22.1% [27]. Alcoholics with advanced liver disease suffer from severe malnutrition, ascites, encephalopathy and, following rupture of the esophageal varices, the complication of severe portal hypertension, followed by upper digestive hemorrhage. In particular, malnutrition is present in a high percentage of patients with ALD, depending on the stage of liver disease, and prognosis is poor [28]. For these patients, nutritional support is essential to avoid progression, as indicated by the guidelines of the European Society for Clinical Nutrition and Metabolism (ESPEN) and the European Association for the Study of the Liver (EASL), which recommend a daily energy intake of 35–40 kcal/kg and a protein intake of 1.2–1.5 g/kg [26]. However, since chronic alcohol ingestion accounts for half of the daily energy intake, in these patients there is a significant energy deficiency caused by reduced food consumption. Thus, metabolic disorders resulting from an insufficient intake of micronutrients, including vitamins and mineral trace elements, are very common in these subjects. Trace elements, such as zinc, copper and iron, are structural cofactors or constituents of key antioxidant enzymes and other metabolic enzymes crucial for the maintenance of homeostasis. Consequently, in alcoholic disease, a deficiency in trace elements will determine a chronic disorder of the antioxidant systems in the long run [29]. Furthermore, the abnormal accumulation of elements such as iron and copper in the liver and/or other organs can also trigger organ malfunction. Several studies have shown how the imbalance of micronutrients, such as zinc, iron and copper along with some vitamins in groups B, D and E, deserves particular attention since their dietary integration in patients with ALD plays a key role in improving pathophysiological and clinical conditions (Table 1).

### 3.1. Vitamin B Group 

Thiamine (B_1_). Deficiency of thiamine is a common feature in chronic alcoholics [36], and it has been considered to be the result of alcoholism, regardless of the underlying liver disease. Malnourished alcoholics should be administered a diet rich in carbohydrates, together with protein-derived calories, ideally via an oral or enteral route. Deficiencies in micronutrients, such as thiamine, are typically encountered in alcoholics and require specific supplementation [37].

Pyridoxine (B6). Lower serum levels of vitamin B_6_ and glutathione have been observed in cirrhosis [30,31] rather than in healthy controls, whereas no significant differences have been found between patients with ALD and those suffering from liver disease of other etiologies [32]. Inadequate levels of vitamin B_6_ could limit glutathione synthesis, affecting the antioxidant capabilities of the liver. Cirrhosis is often associated with increased oxidative stress and decreased antioxidant capacities [38,39]; however, on evaluating the antioxidant effect of a combined supplementation of vitamin B_6_/glutathione in alcohol-related cirrhotic patients, Lai et al. showed that there were no significant effects on oxidative stress indicators [40].

Folate (B9) and cobalamin (B12). Folic acid levels are known to be reduced in patients with liver disease, while levels of vitamin B_12_ are increased [41,42] due to malnutrition and sarcopenia, which are common complications in patients with advanced liver disease, such as stages B and C of the Child-Pugh score [43]. Muro et al. highlighted that plasma levels of folic acid are lower in patients with alcoholic liver disease than in subjects with liver disease of different etiologies [42]. Deficiency of folic acid is one of the most frequently encountered nutritional alterations in ALD patients. Possible causes include the inadequate intake of foods rich in folate, intestinal malabsorption and the actual toxic effect of alcohol itself [44,45]. As a result, it is reasonable to provide folic acid supplements, especially in subjects with ALD [42]. On the contrary, in subjects with alcoholic cirrhosis, plasma levels of vitamin B_12_ are increased [42,45,46,47]. These increases in vitamin B_12_ levels are due to its release from hepatocytes by cytolysis, plus a decrease in clearance due to liver dysfunction [48,49]. However, vitamin B_12_ or folic acid levels do not affect prognosis in cirrhotic patients [42]. 

### 3.2. Vitamin D Deficiency

To date, studies concerning the metabolism of vitamins in the liver have focused mainly on vitamin D. Along with having a well-known effect on bone health and calcium homeostasis, vitamin D also acts as a regulatory factor, modulating cell proliferation, apoptosis, cell-cycle differentiation and the immune system [50]. The accumulated evidence has indicated that people with low vitamin D levels frequently have a higher BMI and have a greater tendency to develop cancer [51,52]. A decrease in vitamin D concentration is very common in liver disease patients; indeed, it plays an important role in hepatic fibrogenesis, suppressing the formation of type I collagen induced by HSC [53]. It has been estimated that 96% of alcoholic patients with both steatohepatitis and/or more advanced disease show reduced serum levels of vitamin D [54]. Trépo et al. reported that severe vitamin D deficiency (<10 ng/mL) was significantly associated with a poor prognosis as well as complications of portal hypertension in cirrhosis [33]. Furthermore, vitamin D supplementation could block the expression of TNFα, a pro-inflammatory cytokine, which further confirms its immunomodulatory role. In a prospective study including patients with alcoholic cirrhosis, it was shown that supplementation with oral vitamin D improved the stage of the *Child-Pugh* score [55].

Overall, these data indicate that vitamin D could also serve as a biomarker in chronic liver disease, regardless of etiology. Given its pleiotropism in the pathophysiological processes inherent to liver fibrosis, a common element of chronicization of liver-related disorders, further studies are necessary to investigate the precise role of vitamin D in ALD and the need for dietary supplementation as a clinical strategy in its prevention and treatment.

### 3.3. Vitamin E Deficiency

Vitamin E has been studied due to its antioxidant properties. The main nutritional sources of vitamin E are vegetables, nuts, olive oil and lean meats. Thanks to its ability to suppress oxidative and inflammatory reactions, vitamin E can slow the progression of atherosclerosis, and it promotes beneficial effects in cancer and diabetes prevention [56,57]. A chronic deficiency could increase oxidative stress; recent studies have shown that a moderate intake of vitamin E is able to modify the composition of the intestinal microbiota, which plays an important role in the pathogenesis of ALD [34]. Vilar-Gomez et al. [58] found that dietary supplementation of vitamin E improves prognosis in liver patients. Throughout the course of cholestatic diseases or drug-induced liver damage, vitamin E protects against hepatocyte necrosis, maintaining mitochondrial integrity [59,60]. Furthermore, in addition to its anti-inflammatory and antioxidant effects, vitamin E is involved in signal transduction and gene expression through the regulation of the P53, NF-kB, and cyclin D_1_ pathways [35]. Vitamin E deficiency has been documented in alcoholic illness due to malnutrition [61,62]. 

Cyclical dietary supplementation could lead to a reduction in serum transaminase levels, an expression of necro-inflammation, due to the suppression of both the inflammatory response and cell apoptosis through the regulation of the EGFR-AKT and EGFR-STAT3 pathways [63].

## 4. Non-Alcoholic Fatty Liver Disease

The term non-alcoholic fatty liver disease, initially coined by Ludwig et al. [64], indicated fatty liver disease without a history of alcohol use, and it is still regarded as the hepatic component of the metabolic syndrome [65,66]. This disease affects almost a quarter of the Western population, primarily obese subjects. To date, there is no approved drug therapy, although different phases of experimentation are under way in clinical trials. Although hepatic steatosis is a benign disease, about 20−30% of patients develop necroinflammation and hepatic fibrosis (non-alcoholic steatohepatitis) with chances of them evolving towards cirrhosis and even into HCC [67,68]. In addition, NAFLD patients are at high risk of developing cardio- and cerebrovascular diseases. Due to the lack of effective therapies and the spread of obesity, post-NASH liver cirrhosis is currently the leading cause of liver transplantation [69]. In fact, obese subjects, especially those presenting co-morbidities, have a low but constant level of systemic inflammation that hinders insulin signaling, leading to insulin resistance (IR), which actively participates in the onset of liver damage [70,71]. Recently, an international group of experts raised concerns about the appropriate use of the term Non-Alcoholic Fatty Liver Disease and proposed to substitute it with Metabolic-Associated Fatty Liver Disease (MAFLD) [72].

The explanation for why some patients with simple steatosis progress to steatohepatitis while others do not has been simplified for many years into the “two-hit” theory, which is based on IR and relatively inert triglyceride deposits within the liver as a cause of the initial damage. However, lipid accumulation in NAFLD occurs as relatively inert TG droplets, and this is now considered a protective mechanism because it prevents the storage of free fatty acids, which are otherwise harmful. Recent evidence points out that inflammation may also precede the accumulation of fat, which would then only become a subsequential response [73,74]. The previously mentioned “two-hit theory” does in fact lead into a “theory of multiple parallel hits” [73] that act synergistically and at different levels in a parallel and non-sequential way and as a result are able to generate NAFLD. The main factors, in addition to the well-known IR [66] and oxidative stress, include adipokines, intestinal dysbiosis, increased gut permeability and exposure to environmental agents [75,76] which interact with each other in genetically predisposed individuals.

The gut microbiota plays an important role in various processes (metabolic, nutritional, physiological and immunological), and it is involved in maintaining health status [77,78]. Its qualitative and quantitative composition varies in the different parts of the intestinal tract and is influenced by different conditions, such as age, dietary habits, ethnicity, way of delivery, exposure to therapies, contact with pathogens and various environmental stimuli [79,80,81,82]. “Dysbiosis” has not only been recognized in gastrointestinal tract diseases (e.g., inflammatory bowel disease) [83], but also in systemic morbidities such as obesity, diabetes mellitus, autism, depression and NAFLD [84]. While the etiological role of the specific gut microbiota has been clearly demonstrated in mice models of NAFLD (e.g., transition from Bacteroides to Firmicutes), the recognition of a corresponding microbiota in humans poses more difficulties.

**Figure 1:**
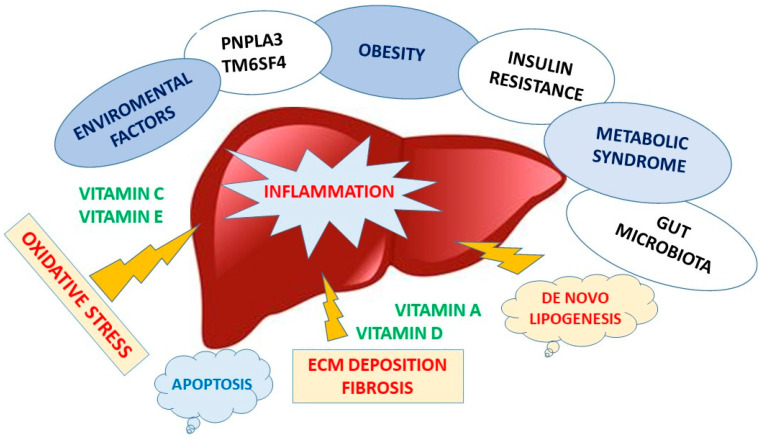
**From NAFLD to NASH: the role of predisposing factors.** Different factors could influence the progression of simple steatosis to steato-hepatitis. The main factors, in addition to insulin resistance and oxidative stress, include adipokines, intestinal dysbiosis, increased gut permeability and exposure to environmental agents, which interact with each other in genetically predisposed individuals (carriers of PNPLA3 and/or TM6SF4 genetic variants). Inflammation triggers fibrobasts activation and ECM deposition resulting in liver tissue remodeling. NAFLD patients tend to have deficiencies in vitamins with antioxidant functions, as C, E, A and D. Thus, vitamin supplementation may represent a support treatment strategy to avoid progression to NASH.

The gut microbiota appears to be involved in the progression from simple steatosis to NASH. In particular, there is an increase in Gram-bacteria, which release LPS penetrating the Kupffer Cells (KC) [85]. It has been hypothesized that endocytosis of LPS by KCs could induce upregulation of cytokine receptors, especially the TNF-α receptor, which also appears to be involved in increasing ROS production [86]. Activated KCs play a role in IR, in the development of fibrosis, in the amplification of inflammation and as a consequence of liver damage (Figure 1).

### 4.1. Role of Micronutrients in Nafld

Some nutraceuticals may play an important role in combatting NAFLD progression when combined with diet and lifestyle changes. Although studies on humans are scarce, anthocyanins and polyphenols seem to have a promising role in the improvement of hyperlipidemia and in the reduction of oxidative stress and vascular inflammation. Some RCTs have demonstrated that Coenzyme Q10 administration (100–300 mg/die) reduces transaminase and gamma-GT levels and the degree of hepatic steatosis, although its administration is limited only to poor oral bioavailability [87]. Various studies have shown that lycopene has a protective role against hepatic inflammation and liver cancer through inhibiting cellular growth and migration both in vivo and in vitro [88].

As has been underlined above, at present there is no specific treatment for NAFLD. International guidelines promote a lifestyle change approach based on a healthy diet. The Mediterranean diet represents the gold standard in preventive medicine, probably due to the harmonic combination of several elements rich in antioxidant and anti-inflammatory properties, hence it is prescribed and recommended in patients with NAFLD as well. Olive oil, the key element of this diet, is associated with benefits against diseases of the cardiovascular system, obesity, diabetes and related metabolic disorders and even cancer [89,90].

The beneficial properties of olive oil have been ascribed to the phenolic compound [91]. Oleocanthal is the phenolic compound of olive oil, which is responsible for its bitter taste, and has strong anti-inflammatory properties based on the inhibition of cyclooxygenase. For this reason, oleocanthal is recognized as a natural non-steroidal anti-inflammatory agent [92]. Several studies have shown that the phenolic compounds of olive oil have beneficial anti-inflammatory, anti-microbial and antioxidant properties [93]. In patients with fatty liver and metabolic syndrome, olive oil supplementation has shown to have multiple beneficial effects on anthropometric and biochemical parameters, including inflammatory cytokines, as well as on abdominal fat distribution [94]. Indeed, recent findings, obtained from in vitro and in vivo studies, have also shown that olive oil has a regulative effect on hepatic lipid metabolism by reducing the lipogenic pathway and thus attenuating liver steatosis [95].

NAFLD patients tend to have deficiencies in vitamins, especially in those with antioxidant functions, such as C, E, A and D. Thus, vitamin supplementation may represent a support treatment strategy to avoid progression to NASH (Table 2).

### 4.2. Vitamin C Deficiency

An inadequate dietary intake could result in a vitamin C deficiency, defined as plasma levels below 23 μM [99]. Ten to twenty percent of the Western population suffers from vitamin C deficiency, which is often associated with an increase in all-cause mortality [12,100,105].

Numerous studies have reported the beneficial administration of multiple vitamins or vitamins together with antioxidant and mineral compounds, but it is hard to identify the role of each single vitamin. In fact, in a meta-analysis including 1225 participants, in which the role of vitamins, as well as β-carotene and selenium, were evaluated, there was no clear evidence to support the efficacy of vitamin C and antioxidants in patients with liver disease or liver cirrhosis [106]. Vitamin C takes part in multiple reactions as a powerful reducing agent and can reduce the formation of mitochondrial ROS, improving the activity of SOD and glutathione peroxidase [107]. Furthermore, it is inversely associated with C-reactive protein (PCR) and with myeloperoxidase, both inflammatory markers [101]. Vitamin C can affect the regulation of adiponectin, which can reduce the accumulation of liver lipids, IR and inflammation and improve oxidative stress [108,109]. In fact, it seems that its deficiency may influence the progression towards NASH [110]. Antioxidant vitamins are reduced in obese and NAFLD patients. Indeed, vitamin C deficiency status is inversely related to BMI and the dietary intake of vitamin C itself, predisposing NAFLD patients to oxidative stress [111,112,113]. Therefore, the link between obesity, NAFLD and oxidative stress suggests that the progression of NAFLD may be accelerated by a vitamin C deficiency and that supplementation could be useful in the treatment of NAFLD [110]. However, according to current evidence, the association between vitamin C deficiency and NAFLD is controversial. As mentioned above, Musso et al. found a significantly lower dietary intake of vitamin C in patients with NASH compared to healthy controls [113]. Han et al. also showed a significant positive association between low vitamin C intake and NAFLD in a male population [114]. In contrast, another study, conducted on a small sample, suggested that both dietary vitamin C intake and plasma vitamin C levels were similar in NAFLD patients and healthy controls [115], and the same conclusions were also confirmed by Madan’s study [116]. A more recent study, conducted on a large sample of 3471 middle-aged and older adults, examined the above-mentioned associations and concluded that dietary vitamin C intake could be inversely associated with NAFLD, especially in a non-obese male population [117]. The gender-based difference could be related to the protective effects of estrogen against NAFLD in females [118], which could weaken the association between dietary vitamin C intake and NAFLD. The literature shows that oxidative stress damage contributes to the progression of NAFLD, and some studies have examined the use of vitamin C and vitamin E supplementation in NAFLD [119], finding no great beneficial effect. Accordingly, the additional supplement of vitamins C and E administered over a 24-month period did not increase the effectiveness of changes in lifestyle (i.e., diet and increased physical activity), which alone led to a significant improvement in liver histology [120]. Thus, although several RCTs have investigated the effect of vitamin C supplementation in NAFLD, the results were inconclusive, as some studies showed that vitamins C and E supplementations were a promising treatment for NAFLD [121] by improving liver fibrosis [122], while others actually showed them to be ineffective [120,123].

### 4.3. Vitamin E Deficiency

Vitamin E levels are consistently low in patients with chronic liver diseases and in particular in NASH, characterized by steatosis and histological findings of lobular inflammation and hepatocyte ballooning.

According to several recent studies, vitamin E appears to be useful in NAFLD treatment: in the TONIC trial, vitamin E seemed to improve hepatocyte ballooning, although it did not improve fibrosis conditions in a pediatric cohort of patients with NASH. In the PIVENS trial, vitamin E reduced hepatic steatosis and lobular inflammation in a group of adult patients with NASH [124,125]. At present, there are no available therapies for NAFLD/NASH, but modifying a patient’s lifestyle, including increasing physical activity and restricting calory intake, represents the cornerstone of NAFLD/NASH therapy. In 2012, the AGA/AASLD introduced vitamin E and the use of pioglitazone as the first-line treatment for histologically documented NASH with or without diabetes, although the long-term efficacy of this treatment is yet to be demonstrated [126]. It is well known that oxidative stress is a major factor in NASH progression to liver cirrhosis; vitamin E is a free radical scavenger. The current literature reports that a one-year treatment with vitamin E reduces transaminase and TGF-1 levels in adult patients with NASH that do not respond to diet therapy [119,121,126]. The PIVENS trial demonstrated the efficacy of vitamin E (800 mg/die for 2 years) versus placebo on NASH histology improvement, observed in NASH adult patients without diabetes and cirrhosis. Reductions in transaminase values and improvements in steatosis and inflammation were also observed [127]. A study by Sumida et al., demonstrated that long-term treatment with vitamin E (300 mg/die) for more than 2 years could improve hepatic fibrosis in NASH patients, especially in the presence of increased levels of hepatic cytolysis enzymes and proven insulin resistance [128]. A recent meta-analysis reported that in NAFLD/NASH patients, vitamin E reduces the values of liver enzymes compared with placebo and improves histological parameters, and it could therefore be considered a promising treatment in patients with NAFLD and raised transaminases; nevertheless, further studies are needed [129] to confirm this.

Moreover, the protective role of vitamin E has been demonstrated in animal models in which a deficiency of both vitamin E and selenium was related to oxidant agent activation, along with abnormalities in the metabolome and hepatic transcriptome, leading to cellular death and progression to NAFLD [103]. In line with these findings, it has also been demonstrated that a combined approach with both pioglitazone and vitamin E may reduce liver cirrhosis and the number of HCC cases and thus reduce the need for liver transplantation [104]. However, the current literature also reports that long-term use of vitamin E at elevated doses can lead to potential toxic effects and can be related to an increased mortality risk for all causes, as well as for prostate cancer and hemorrhagic stroke [126,130]. 

### 4.4. Vitamin A Inadequacy 

Fat-soluble vitamin A (retinyl esters) is stored in HSCs in large droplets of lipids during their resting state. In this regard, it should be remembered that lipids play a dual role: on the one hand, HSCs with a lipid-rich state are indicative of a healthy liver, but on the other, lipid-laden hepatocytes also obstruct liver function, resulting in hepatic fibrosis. Paradoxically, in response to hepatocyte lipid accumulation, HSCs lose their large vitamin A-laden lipid droplets, turning into a phenotype producing extracellular matrix (ECM) that ultimately results in liver fibrosis [96]. One of the first studies on vitamin A in animal models showed that rats subjected to a nutritional restriction of vitamin A for eight weeks did not show the large lipid droplets typical of HSCs. Furthermore, in studies on mice lacking the lecithin-retinol acyltransferase enzyme (LRAT), which esterifies retinol, it was seen that very few retinyl esters were stored in the liver [131]. On the basis of these premises, it appears that NAFLD is triggered and aggravated by a series of factors correlating with hepatic necroinflammation (adipokines/cytokines) [132]. It is not yet clear how the metabolism of hepatic vitamin A can affect inflammation, oxidative stress, the development of fibrosis and cancer and the increased risk of NAFLD, although some genetic variants in retinol metabolism are known to be associated with NAFLD and therefore disease progression. In fact, in two mice models of NAFLD, an impaired metabolism of retinol was found, resulting in the accumulation of vitamin A in hepatocytes and consequently progression of disease [97]. Moreover, a cross-sectional analysis demonstrated the correlation between serum levels of retinol, vitamin A and other antioxidants with hepatic fibrosis in NAFLD, evaluated by FibroScan. In fact, a high percentage of serum retinol deficiency was correlated with advanced liver fibrosis and significant vitamin A deficiency [98].

### 4.5. Vitamin D Deficiency

A correlation has been shown between vitamin D deficiency and some features of metabolic syndrome, such as IR and dyslipidemia [133]. Studies using a rat model of NAFLD have also shown that vitamin D deficiency exacerbates hepatic inflammation, increasing the activity of toll-like receptors (TLR) and IR [134]. In particular, a cross-sectional study in two Italian cohorts suggested that low 25-(OH)-D levels were associated with advanced hepatic steatosis and fibrosis in patients with NAFLD [99]. Additionally, some recently published studies comparing serum vitamin D levels in NAFLD patients with different histological severity grades showed conflicting results, since low vitamin D levels did not appear to be associated with higher stages of fibrosis [135,136]. This may depend on how hepatic fibrosis was assessed in the studies. In fact, it should be emphasized that even if liver biopsy is the gold standard for diagnosing liver damage and the relative stage, it is an invasive procedure and is not applicable on a large scale; therefore, a lot of studies use noninvasive markers to grade fibrosis.

In a study on a significant population sample, comprising 6800 patients, a reverse relationship between serum alanine transaminase (ALT) and 25-(OH)-D levels was found [137]. In a Korean study, 3878 adolescents (including 78.9% with suspected NAFLD, defined as an increased alanine transaminase concentration >30 U/L) presented hypovitaminosis D (25-hydroxyvitamin D levels < 20 ng/mL); therefore, adolescents with vitamin D deficiency were considered at increased risk of NAFLD [138]. However, other studies have not supported the relationship between NAFLD severity and 25-(OH)-D levels [139]. Observational studies showed that 25-(OH)-D supplementation did not have any significant effect either on histopathology or on transaminase levels [140]. A randomized, double-blind study analyzed the effects of vitamin D supplementation (2000 IU per day for 24 weeks) in 65 patients with Type-2 diabetes mellitus and NAFLD. The outcome showed that the hepatic fat fraction, measured by MRI, did not decrease from baseline to end of treatment; neither did serum transaminases, CK18-M30, N-terminal pro-peptide procollagen III levels (P3NP) and fatty liver index (FLI); and nor did metabolic (fasting glycemia, HbA1c, lipids, HOMA-IR, HOMA)-β, ADIPO-IR, body fat distribution) and cardiovascular (ankle-arm index, intima-media thickness, flow-mediated dilation) parameters [141]. 

Kitson MT et al., while studying patients with NASH, analyzed their histopathological aspect before and after six months of treatment with 25,000 IU of cholecalciferol and recorded no evidence for its reducing inflammation and fibrosis or intra-hepatocyte fat [142]. A similar result was found in a study of 120 patients re-evaluated by ultrasound following calcitriol supplementation compared to placebo [143]. Only one RCT tested the safety and efficacy (assessed with the NAFLD Activity Score) of oral supplementation at 800 UI per day of cholecalciferol in combination with docosahexaenoic (DHA) in children with NASH (diagnosed by biopsy) [144]. However, these findings could be attributed more to the DHA treatment than to vitamin D [120]. It is interesting to note that an increase in vitamin D serum levels was observed in a study conducted by Seung Min Lee et al. in 82 patients with a new diagnosis of NAFLD subjected to caloric restriction for two months, and there was a consequent improvement in NAFLD parameters; therefore, weight loss proved to be more effective than vitamin supplementation [145], the lower vitamin D levels corresponding to an increased risk of abdominal obesity and metabolic syndrome.

Finally, a clear, direct correlation between vitamin D and NAFLD severity has not yet been demonstrated. Further larger and higher quality studies are also needed to better understand the mechanisms by which vitamin D and its relative receptor is involved in the gut−liver axis [146].

## 5. Advanced Liver Disease: Cirrhosis and HCC

Liver cirrhosis is a chronic and degenerative disease of the liver of various etiology and is a significant cause of morbidity and mortality. In Italy, there are about 100,000 cirrhotic patients, and the correlated deaths attributable to liver cirrhosis, along with its most fearsome complication, HCC, are about 20,000/year, according to the latest official ISTAT data. Cirrhosis leads to a complex clinical picture, and the most common complications are ascites, gastro-esophageal varices with hemorrhage, hepatic encephalopathy and finally development of HCC, liver failure and death. HCC accounts for over 90% of primary liver cancers and is currently the sixth most common cancer in the world: according to the latest data, there are approximately 841,000 new cases each year and 781,000 deaths [147]. Liver cirrhosis is the most important risk factor for HCC, sharing several causal factors, such as HCV and HBV chronic infection, ALD, post-NASH cirrhosis, hemochromatosis and chronic autoimmune liver disease. Patients with cirrhosis and HCC are deficient in several micronutrients and vitamins (Table 3). Specifically, in liver cirrhosis, there is a marked prevalence of low vitamin A levels, which correlates with liver dysfunction [148].

### 5.1. Vitamin A Deficiency

Reduced levels of vitamin A have usually been found in patients with chronic viral hepatitis, and low levels of serum retinol have also been detected in patients with cirrhosis and HCC [157]. From this evidence, it would seem that a lack of vitamin A is primarily associated with liver fibrosis development, because of retinoid content loss in the HSCs, after their activation and extracellular matrix deposition [149]. A recent study on patients with advanced liver disease showed a high prevalence of vitamin A and D deficiencies, and that vitamin A is strongly correlated with liver dysfunction, portal hypertension and levels of bile acids [148]. Studies on animals with primary biliary cirrhosis and treated with urso-deoxycholic acid and vitamin A, found that liver parenchyma histology improved and that the pool of bile acids was reduced [158]. However, it is not yet clear whether vitamin supplementation has a beneficial effect, and a potential important limitation in the large-scale use of vitamin A supplementation in clinical practice is its possible hepatotoxicity [159]. In fact, it has been reported that integration of vitamin A, in particular the synthetic type, could be hepatotoxic at certain doses, causing cirrhosis and veno-occlusive disease [160].

### 5.2. Vitamin D Deficiency

A marked prevalence of vitamin D deficiency has also been reported in patients with liver cirrhosis. In fact, vitamin D has an anti-fibrotic effect on HSC through VDR, which activates signal transduction pathways, resulting in a silencing of the expression of pro-fibrogenic genes [15]. In a study carried out on patients with genetic variants affecting vitamin D levels in chronic liver disease, it was found that those with reduced levels of 25 (OH)-vitamin D had more advanced stages of hepatic fibrosis (assessed by Fibroscan) and that hypovitaminosis D had a more significant impact on the onset than on the progression of liver fibrosis [150]. Similarly, another study of genetic variants affecting vitamin D levels identified the variant DHCR7 GG, an independent risk factor for severe fibrosis, which is also associated with lower serum vitamin D levels [161]. A recent study on 100 Caucasian patients with liver cirrhosis showed that low vitamin D levels were inversely correlated with the Child Pugh score and IL-6 levels and directly proportional to vitamin D binding protein (VDBP) values. It was also found that they determined the severity of cirrhosis and mortality [162]. However, the latter evidence is in contrast with other studies, which reported no relationship between vitamin D levels and liver fibrosis [163]. As for vitamin D supplementation in this category of patients, a systematic review concluded that taking vitamin D does not appear to impact liver-related morbidity and quality of life [6]. Significantly, lower levels of vitamin D were reported among patients with autoimmune diseases, such as primary biliary cirrhosis (PBC) or autoimmune hepatitis (AIH). A recent study on 79 patients found vitamin D deficiency in PBC patients with severe liver damage and fibrosis or with concomitant immune-mediated disorders, suggesting its possible role as an advanced disease marker. Furthermore, this study also showed increased levels of vitamin D in patients treated with ursodeoxycholic acid [164]. Another study on AIH patients reported that a severe vitamin D deficiency was associated with treatment non-response and was also an independent risk factor of developing cirrhosis, liver-related mortality or liver transplant requirement. Conversely, patients whose vitamin D levels were corrected by supplementation obtained a complete treatment response and outcomes improved [165]. Thus, in the field of advanced liver disease, more detailed research is needed regarding vitamin D supplementation because there are many evaluation biases within existing studies and a low quality of evidence.

### 5.3. Vitamin E Deficiency

Several studies have reported low levels of vitamin E in cirrhosis and a high risk of developing HCC. In particular, tocopherol and other liposoluble vitamin levels seem to be lower in both plasma and liver tissue in cirrhosis-associated HCC, whereas normal or even raised tocopherol levels are associated with liver metastasis from digestive-tract neoplasms. HCC nodules show a severe reduction in tocopherol levels compared to normal liver or cirrhotic liver tissue. This is probably due to the cholestasis associated with advanced cirrhosis and HCC, as well as to a lower intestinal absorption of tocopherol [151]. 

### 5.4. Vitamin K Insufficiency

For many years, it was believed that in cirrhosis there was an imbalance between coagulation factors and an increased risk of bleeding, worsened by a low platelet count [166]. In fact, patients with cirrhosis present hemostatic alterations secondary to the reduced availability of pro-coagulant and anti-coagulant factors. The net effect of these changes is a rebalanced hemostatic system. The Italian Association for the Study of the Liver (AISF) and the Italian Society of Internal Medicine (SIMI) promoted a Consensus Conference on Hemostatic Balance in patients with cirrhosis [167]. The role of vitamin K deficiency in the coagulopathy of liver disease is controversial; indeed, the benefits of vitamin K administration in liver disease have been questioned by several studies. Vitamin K deficiency supports liver injury; however, its deficiency is determined by many factors, such as intra- and extra-hepatic cholestasis, prolonged oral antibiotic therapy, malnutrition and malabsorption. Vitamin K is commonly prescribed for patients with end-stage liver disease, but it has been shown to be effective only in correcting abnormalities in coagulation parameters in cases of biliary tract disease and gut sterilization by broad-spectrum antibiotics, in which vitamin K deficiency is the main reason behind the coagulopathy [168]. By contrast, in patients with liver cirrhosis and persistent liver damage, Saja et al., demonstrated that vitamin K administration does not affect coagulation parameters or risk of bleeding [169].

The role of vitamin K in HCC deserves a more thorough discussion. When vitamin K is insufficient, vitamin K-dependent proteins are undercarboxylated and become functionally defective proteins known as PIVKA-II (protein induced by vitamin K absence-II), resulting in coagulopathy and bone disease [170,171]. Traditionally, prothrombin time (PT) has been used as an indicator of vitamin K status. The current literature shows that PT is a non-sensitive marker of vitamin K status because PT values change when prothrombin (factor II) decreases to 50% of normal. Furthermore, vitamin K_1_ levels do not reflect stored vitamin K but only recent vitamin K intake. Some recent studies have shown that PIVKA-II levels are a more sensitive marker of vitamin K status. Furthermore, PIVKA-II is a well-known potential diagnostic marker for HCC detection; several studies have confirmed that serum PIVKA-II levels may improve the early detection of HCC and tumor prognosis, especially in HBV-related HCC [167,168,169]. According to some studies, PIVKA-II appears to induce the over-expression of vascular endothelial growth factor (VEGF), enhancing vascular invasion. In fact, increased levels of PIVKA-II have been detected in patients with HCC and microvascular metastasis. For these reasons, PIVKA-II is indeed a useful marker for predicting microvascular invasion [154].

Vitamin K insufficiency, confirmed by elevated PIVKA-II levels, is very common in cholestatic liver diseases such as Alagille syndrome, biliary atresia and primary biliary cholangitis. Two recent studies have shown that vitamin K deficiency, assessed by elevated PIVKA-II levels, affects at least two-thirds of children and adults with cholestatic liver diseases, despite oral supplementation [170,171]. These studies have demonstrated that vitamin K deficiency is directly related to disease severity, especially in children. Moreover, even vitamin K supplementation to the point of normalizing PT levels and coagulation may not be sufficient to maintain appropriate levels of carboxylate osteocalcin, so that children with cholestatic diseases are at risk of osteopenia and osteoporosis [170]. 

Vitamin K_2_ and its derivates have been demonstrated to have anti-proliferative effects in hepatoma cells. Moreover, a recent study reported the efficacy of vitamin K_2_, in combination with other anti-tumoral agents, on disease recurrence and long-term survival in patients with HCC after resection or percutaneous local ablation therapy [155,156]. Further studies showed that vitamin K helps to prevent HCC [172,173]. It is also known that the development of portal vein invasion is related to levels of PIVKA-II. Otzuka et al. reported that K_2_ inhibited the growth of HCC cells through the suppression of cyclin D_1_ expression [174]. Since vitamin K deficiencies have been observed in HCC patients, along with increased PIVKA-II levels, the use of vitamin K supplementation has been studied in cancer cell lines, including HCC. Anti-proliferative effects have been shown in vitro, though they have not yet been demonstrated in vivo. Furthermore, it has been shown that vitamin K_2_ analogs may suppress the development of HCC in women with viral-related cirrhosis [155,175]. Some studies have also described a role for vitamin K combined with Sorafenib in inducing growth inhibition and apoptosis in HCC cells (compared to patients treated with Sorafenib alone) by extending both progression-free survival and overall survival [176].

## 6. Liver Transplantation 

As could be expected, micronutrient deficiency is widespread in patients with chronic liver disease, and this is particularly common for vitamin D in most patients at the time of transplantation, although it is not yet clear whether this represents a risk factor for post-transplant infections [177]. Bitetto et al., assuming that vitamin D acts as an immunomodulator in their experimental transplantation, examined 133 transplanted patients and concluded that vitamin D could promote immune tolerance towards liver allografts [178]. In a recent study on a small sample of post-liver transplantation patients who received supplements of cholecalciferol (vitamin D_3_) at 2500 units per day for 12 weeks, 78% of them benefited from the treatment, with a decrease in the incidence of transplantation rejection as well as infections in these immuno-compromised patients [179]. More recently, another study confirmed the above-mentioned evidence of the beneficial role of Vitamin D supplements, which is associated with a lower risk of acute rejection and infections and promotes immune tolerance towards liver allografts through the involvement of regulatory T cells [180] and finally, by reducing hepatocyte apoptosis [181,182]. Moreover, since liver transplant patients, in addition to having pre-existing hypovitaminosis D, are subjected to treatment with corticosteroids and immunosuppressants, they can be at greater risk of osteoporosis and fractures. Therefore, supplementation therapy with vitamin D could be a reasonable approach [183,184]. However, another retrospective study, despite confirming the prevalence of moderate to severe vitamin D deficiency in these patients, was not able to support a correlation between this deficiency and worsening bone conditions and/or functional outcomes after liver transplantation [185], thus making further studies necessary.

Both vitamin A and E have also been found to be deficient and linked to post-liver transplantation reperfusion injury, and it appears that Child-Pugh class, bilirubin levels and elevated BMI may predict vitamin A deficiency [186]. A retrospective study found a prevalence of low serum levels of vitamins A, D, E and zinc in adult patients evaluated for liver transplantation (vitamin A 77%, D 63%, E 37%), and these deficiencies correlated with higher Child-Pugh scores [187]. At present, there are few other studies aimed at establishing correlations between the hypovitaminosis described and clinical outcomes. In addition, since vitamin K is often deficient, bleeding can be exacerbated due to the hypocoagulability it causes in addition to the collateral circulation caused by portal hypertension and the increase in fibrinolysis that may occur during this surgical procedure [188]. 

Finally, it was recently found that ascorbic acid is able to reduce fibrinolysis and improve clot rigidity in liver transplantation recipients, and therefore, it can be considered as a strategy during transplantation [189]. However, further and larger studies are needed to confirm the role of vitamin supplementation in liver transplantation candidates.

## 7. Conclusions

As this review has shown, vitamin deficiencies are very common in chronic liver disease, especially in the advanced stages, and supplementation strategies can help to improve the redox and immunity state of patients. In particular, vitamin D is important because of its immuno-modulator role in improving viral response in patients with chronic hepatitis, but also in reducing inflammation in NAFLD and in reducing the incidence of transplantation rejection and infections in immuno-compromised patients. 

Other vitamins, whose roles are of note in liver metabolism, are vitamins C and E. These act as antioxidants, and their role in the progression of NAFLD to NASH is indeed relevant. Adding vitamin E to pioglitazone in diabetic patients with histologically documented NASH has become the first line of treatment, and it has been incorporated in the AASLD guidelines [2]. PIVKA-II, a defective vitamin K protein, is a diagnostic marker in cirrhotic-HCC patients; moreover, vitamin K has an anti-proliferative effect, leading to a reduction in disease recurrence and better long-term survival in HCC patients, when combined with anti-tumoral agents.

In conclusion, the role of vitamins should not be underestimated when approaching liver diseases, and supplementation strategies should be part of a successful treatment policy.

## Figures and Tables

**Table 1:** Trace elements and vitamins imbalance in ALD.

	Status in Liver Disease	Physiological Role	Potential Role in Liver Disease
Zinc	↓	Neurotransmitter functions, intracellular signaling transduction, inflammatory response, ROS production, immune regulation, wound healing, gene expression	Mitochondrial dysfunction, oxidative injury, glutathione depletion [29]
Iron	↑	Transportation of oxygen, DNA and ATP synthesis	HSCs activation, liver fibrosis promotion, ferroptosis, increased risk of infections, ROS increased production [29]
Copper	↓/↑	Bone marrow and CNS homeostasis; co-factor of antioxidant enzymes	Interaction with other trace elements [29]
Vitamin B group	↓	Pleiotropic co-enzymatic activity, direct precursor for metabolic substrates, antioxidant response	*Vitamin B6:* limitation of glutathione synthesis affecting antioxidant capability of the liver [30,31,32]
Vitamin D	↓	Calcium homeostasis immuno-modulating activity	Vitamin D deficiency is associated with poor prognosis and complications of portal hypertension in cirrhosis [33]
Vitamin E	↓	Antioxidant immuno-modulating activity	Deficiency could increase oxidative stress, modifying the composition of gut microbiota [34] in addition to anti-inflammatory and antioxidant effects and signal transduction of P53, NFkB and Cyclin D1 pathways [35]

Note: ↑—means increased; ↓—means reduced.

**Table 2:** Enzymatic and non-enzymatic antioxidants and their role in NAFLD.

Antioxidants	Status in Liver Disease	Potential Role in Liver Disease
**Enzymatic**		
Superoxide dismutase (SOD)		Possible reduction of inflammatory-induced liver damage [21]
Glutathione		
**Non-enzymatic**		
Flavonoids	↓	Reduction of IL-1α and IFN-γ
Lycopene	↓	Inhibition of liver diseases, including NAFLD and liver cancer [88]
Coenzyme Q10	↓	Reduction of NAFLD degree, transaminases, gamma-GT levels, oxidized LDL levels [87]
Olive oil (phenolic component)	↓	Reduction of lipogenic pathway and thus attenuation of liver steatosis [95]
Vitamin A	↓	IFN response modulation Fibrosis development [96,97,98]
Vitamin C	↓	Possible influence on the progression towards NAFLD [99,100,101]
Vitamin D	↓	Reduction of fibrosis in HCV-infected patients, reduction of inflammation in NAFLD and HCC proliferation [102]
Vitamin E	↓	Reduction of LFTs in NAFLD/NASH patients, liver steatosis and lobular inflammation. Possible cirrhosis and HCC reduction if combined with pioglitazone in NAFLD [103,104]

Note: ↓ means reduced.

**Table 3:** Role of vitamin deficiencies in advanced liver disease and HCC.

	Status in Liver Disease	Potential Role in Liver Diseases
Vitamin A	↓	Fibrosis development, if deficient [148,149]
Vitamin D	↓	Anti-fibrotic effect [15] Reduction of HCC proliferation [150]
Vitamin E	↓	Possible HCC reduction [151]
Vitamin K		PIVKA-II as a diagnostic marker in HCC patients [152,153,154] Reduction of disease recurrence and long-term survival in patients with HCC after resection or percutaneous local ablation therapy when combined with anti-tumoral agents [155,156]

Note: ↓ means reduced.
